# Characterization of *Culex pipiens* cell lines: virus infection and RNAi response

**DOI:** 10.1186/s13071-026-07248-w

**Published:** 2026-01-28

**Authors:** Sarah Gothe, Swati Jagtap, Philipp Böhmer, Melinda Reuter, Svea Frank, Vattipally B. Sreenu, Lesley Bell-Sakyi, Andres Merits, Mine Altinli, Esther Schnettler

**Affiliations:** 1 Bernhard-Nocht Institute for Tropical Medicine, Hamburg, Germany; 2 German Center for Infection Research (DZIF), Partner Site Hamburg-Luebeck-Borstel-Riems, Hamburg, Germany; 3 Faculty of Mathematics, Informatics and Natural Sciences, University of Hamburg, Hamburg, Germany; 4 MRC-University of Glasgow-Center for Virus Research, Glasgow, G61 1QH UK; 5 Department of Infection Biology and Microbiomes, Institute of Infection, Veterinary and Ecological Sciences, University of Liverpool, Liverpool, L3 5RF UK; 6 Institute of Bioengineering, University of Tartu, Tartu, Estonia

**Keywords:** *Culex*-borne arbovirus, RNAi, *Culex pipiens*-derived cell line, Insect-specific virus, Arbovirus

## Abstract

**Background:**

Arboviruses transmitted by mosquitoes pose a global health threat, causing diseases ranging from mild fevers to severe encephalitis and hemorrhagic fevers. Despite their growing impact, arbovirus research is hindered by biosafety constraints and the need of specialized BSL-3 insectariums. To circumvent these challenges, mosquito-derived cell lines have become indispensable tools for investigating virus-vector interactions. However, most available cell lines originate from *Aedes* and *Anopheles* spp., creating a critical research gap for other key vectors such as *Culex* spp. Although a few cell lines were previously established, they did not represent primary transmitters of West Nile virus (WNV) and other emerging arboviruses in Europe, such as *Culex pipiens*.

**Methods:**

To address this gap, the current study aimed to characterize two recently established *Culex pipiens* cell lines: CPE/LULS50 (*Culex pipiens pipiens* & *molestus*) and CPL/LULS56 (*Culex pipiens molestus*) in more detail including testing their virus susceptibility, antiviral RNAi response, and possible presence of insect-specific viruses.

**Results:**

The replication of arboviruses from three clinically relevant families (*Flaviviridae, Peribunyaviridae*, and *Togaviridae*), as well as insect-specific viruses, was observed in both CPE/LULS50 and CPL/LULS56 cell lines. Furthermore, small RNA profiling revealed production of virus-specific small interfering RNA (siRNA) in both cell lines for all tested viruses. Interestingly, virus-specific PIWI-interacting RNA (piRNA) was only detected for the *Peribunyaviridae.*

**Conclusions:**

The current study demonstrates that the CPE/LULS50 and CPL/LULS56 cell lines are suitable candidates to facilitate research into *Culex*-specific virus-vector interactions, ultimately contributing to mitigation of the impact of *Culex*-borne arboviruses on public health.

**Graphical abstract:**

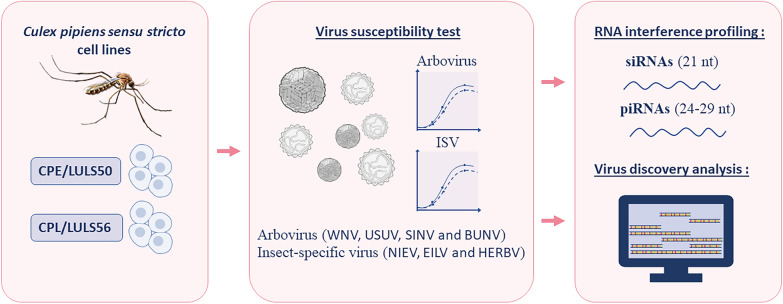

**Supplementary Information:**

The online version contains supplementary material available at 10.1186/s13071-026-07248-w.

## Background

Arboviruses (arthropod-borne viruses), primarily transmitted by mosquitoes and other arthropods, pose a major global health threat. They can cause a range of diseases in humans and animals, from mild febrile illness to severe encephalitis and hemorrhagic fevers [1]. With climate change, urbanization, and the expanding geographical range of mosquito vectors, the frequency and intensity of arboviral outbreaks have increased [2, 3]. Public health interventions remain limited, partly due to the unpredictability of mosquito populations and the complexity of vector control. *Culex pipiens *sensu stricto (*ss*) mosquitoes are major vectors of West Nile virus (WNV) and Usutu virus (USUV), two flaviviruses of increasing concern in Europe and other parts of the Northern Hemisphere [4–8]. However, to design effective strategies for disease prevention, a deeper understanding of virus-vector interactions is essential.

Mosquito-derived cell lines provide valuable in vitro systems for studying virus–host interactions. They have been instrumental in elucidating how arboviruses replicate and interact with their insect hosts [8]. A critical aspect of these arbovirus–vector interactions is the mosquito’s immune system. RNA interference (RNAi), a sequence-specific RNA degradation mechanism, is considered a major antiviral immune response in mosquitoes [9]. Here, the RNAi system comprises three major pathways: small interfering RNA (siRNA), PIWI-interacting RNA (piRNA), and microRNA (miRNA). Among these, the exogenous siRNA pathway is thought to be the primary antiviral mechanism, while the antiviral roles of miRNA and piRNA pathways are less clear. The exogenous (exo-) siRNA pathway is initiated when the Dicer-2 enzyme cleaves viral double-stranded (ds)RNA into 21-nucleotide virus-derived small interfering RNAs ((v)siRNAs), which are then incorporated into the RNA-induced silencing complex to guide the degradation of complementary viral RNA [10, 11]. Unlike the siRNAs, piRNAs are 24–30 nucleotides long, generated without the involvement of Dicer, and linked with Argonaute proteins from the PIWI family [12]. In mosquitoes, the interaction of the exo-siRNA and piRNA responses with arthropod-borne viruses has been shown by the detection of vsiRNAs and vpiRNAs [13]. In addition to arboviruses, mosquitoes harbor insect-specific viruses (ISVs), which replicate exclusively in insect hosts and cannot infect vertebrates [14, 15]. ISVs are widespread across mosquito species and are commonly found in mosquito-derived cell lines [15–18]. Persistent ISV infections have been reported to be able to modulate host immunity, including the RNAi machinery, and influence the replication of co-infecting arboviruses [4, 14, 19, 20].

Even though mosquito-derived cell lines provide valuable in vitro systems for studying virus–host interactions, most available cell lines originate from *Aedes* and *Anopheles* spp., such as the well-characterized C6/36, U4.4 and Aag2 cells, leaving a gap in research tools for other relevant vectors, particularly *Culex pipiens ss* [21–24]. While other *Culex* spp. cell lines exist, such as those derived from *Culex tarsalis* (Ct) and *Culex quinquefasciatus* (HSU), *Culex pipiens ss*-derived cell lines remain underrepresented.

Recently, *Culex pipiens ss* cell lines have been established: CPE/LULS50 (from *Cx. pipiens* biotype *pipiens* and biotype *molestus*), CPL/LULS56 (biotype *molestus*) [25], and CpE3 (*Cx. pipiens* biotype *pipiens*) [26]. However, these lines have not yet been fully characterized regarding their RNAi competency, susceptibility to (arbo)viral infection, and presence of persistent ISVs. This knowledge is important to enable the use of these cells to study virus–host interactions in more detail and opens up the possibility to study possible differences in this interaction between all available Culex-derived cell lines.

In this study, we assessed the potential of the CPE/LULS50 and CPL/LULS56 cell lines as in vitro systems for studying virus–vector interactions. We first evaluated their susceptibility to representative arboviruses and ISVs, including Bunyamwera orthobunyavirus (BUNV), peribunyavirus Herbert virus (HERBV), flaviviruses WNV, USUV, Niénokoué virus (NIEV), and togaviruses Sindbis virus (SINV) and Eilat virus (EILV). Using small RNA sequencing, we then investigated their RNAi responses, analyzing production of virus-derived siRNAs and piRNAs. Lastly, the same datasets were screened for evidence of persistent ISV infections. Our results contribute to the recognition of these cell lines as models useful for studying virus–host interactions in *Culex pipiens ss*.

## Methods

### Cell lines

The CPE/LULS50 cell line consists of *Cx*.* p*. biotype* pipiens* and biotype *molestus* embryo-derived cells, and the CPL/LULS56 cell line consists of *Cx*.* p*. biotype *molestus* larva-derived cells [25]. The *Aedes albopictus* cell line C6/36 was received from A. Kohl (University of Glasgow Centre for Virus Research [CVR], Glasgow, UK) [27, 28].

All mosquito cell lines were maintained at 28 °C in L-15 (Leibovitz) medium (Thermo Fisher Scientific Inc., Waltham, MA, USA) supplemented with 10% tryptose phosphate broth (TPB; Gibco Life Technologies), 100 units/ml penicillin, 100 µg/ml streptomycin (ThermoFisher Scientific), and either 10% fetal bovine serum (FBS; ThermoFisher Scientific) for C6/36 cells or 20% FBS for CPE/LULS50 and CPL/LULS56 cells.

As mammalian cells, the human HuH-7 cell line [29] and the baby hamster kidney cell line BHK-21 (ATCC CCL-10, [30]) were maintained at 37 °C with 5% CO_2_ in Glasgow Minimum Essential Medium (GMEM; ThermoFisher Scientific) supplemented with 5% FBS, 10% TPB (Gibco Life Technologies), 100 units/ml penicillin, and 100 µg/ml streptomycin. Vero cell line (ATCC CCL-81) was maintained at 37 °C with 5% CO_2_ in Dulbecco’s Modified Eagle Medium (DMEM, PAN-Biotech GmbH) supplemented with 10% FBS, 10% TPB, 100 units/ml penicillin, and 100 µg/ml streptomycin.

### Viruses

BUNV (segment S accession number NC_001927, segment M accession number NC_001926, segment L accession number NC_001925) stock was produced as described elsewhere [31]. Briefly, BUNV stock was produced in BHK-21 cells, the supernatant was harvested upon the detection of cytopathic effect (CPE), and cell debris was removed by centrifugation. Virus titer was determined via plaque assay on BHK-21 cells with 1.2% Avicell as overlay.

USUV 491 (accession number KY426758) and WNV lineage 2 (WNV2; accession number MH924836.1) [32, 33] stocks were produced in Vero cells. Virus titer was determined via tissue culture infectious dose 50 (TCID_50_) assay on Vero cells for USUV and HuH-7 cells for WNV2. Shortly, 4 × 10^4^ cells per well in 180 μL cell medium were seeded in a 96-well plate and incubated for 24 h. Cells from the first column were then inoculated with 20 μL of virus stock solution, and a serial dilution (1:10) was performed from this point on. After incubation and onset of CPE, cells were fixated with 8% formaldehyde for 60 min, and stained by removal of formaldehyde and addition of 100 μL of crystal violet solution for 60 min. TCID_50_ was calculated using the Spearman–Kärber algorithm as described [34–36].

SINV TOTO1101 strain was recovered from infectious cDNA clones, as previously described [37]. Briefly, the plasmid was linearized with XhoI, purified, and in vitro transcribed using the SP6 RNA Polymerase (Ambion, Invitrogen) in the presence of a cap analogue. The resulting RNA was transfected into BHK-21 cells, and upon observation of CPE, the supernatant was collected and used to infect fresh BHK-21 cells in a T75 flask. Upon CPE observation, the supernatant was harvested, clarified by centrifugation, and virus titer was assessed via TCID_50_ assay on BHK-21 cells.

EILV (accession number JX678730.1), NIEV (accession number NC_024299.2), and HERBV (accession number NC_038714; NC_038713; NC_038712) stocks were produced in C6/36 [15, 17, 38]. EILV and NIEV virus titer was assessed via TCID_50_ assay in C6/36 cells by immunofluorescence detection of dsRNA using the monoclonal antibody 3G1 [39], followed with the secondary goat anti-mouse immunoglobulin (Ig)G (H + L) Alexa Fluor 488 (Invitrogen). HERBV virus titer was assessed via TCID_50_ assay in C6/36 cells by immunofluorescence detection of anti-HERBV-N (rabbit polyclonal), followed by with donkey anti-rabbit IgG (H + L) Alexa Flour 594 (Invitrogen). DNA were labelled with DAPI stain.

### Virus growth kinetics

For the virus growth kinetics, a total of 6.5 × 10^5^ of CPE/LULS50 or CPL/LULS56 cells were seeded per well, in duplicate, in a 6-well plate and incubated overnight at 28 °C. Approximately 24 h post-seeding, the cells were infected with BUNV, SINV, WNV, or USUV at a multiplicity of infection (MOI) of ten calculated accordingly to the number of seeded cells. For the ISVs (EILV, NIEV, and Herbert virus), MOI of 1, calculated accordingly to the number of seeded cells, was used. Shortly, total SN was removed and infections were performed in 500 µL of supplemented L-15 medium per well. Cells were incubated for 1 h at 28 °C and subsequently total SN was replaced with 2 mL of fresh supplemented L-15 medium and immediately sampled for the timepoint 0 days post-infection (dpi). The supernatant was further sampled at 1 dpi, 2 dpi, and 3 dpi for all viruses. Additional samples were collected on day 6 post-infection for all arboviruses, and day 8 post-infection for USUV and WNV. For all samples, a TCID_50_ assay (as described for the corresponding virus stock production) was performed to assess the virus titer of three independent biological replicates. The data were log-transformed and the mean and SEM of the log-transformed data were represented in a graph.

### Small RNA sequencing and analysis

For small RNA sequencing, a total of 6.5 × 10^5^ of CPE/LULS50 or CPL/LULS56 cells per well were seeded in a 6-well plate in duplicate (replicate A and B), infected with SINV, BUNV, or WNV (MOI 10) and incubated at 28 °C for 48 h for SINV and BUNV, and 96 h for WNV. Total RNA was isolated using TRIzol (Thermo Fisher Scientific Inc., Waltham, MA, USA) with glycogen as a carrier, according to the manufacturer’s instructions.

Library preparation and Illumina-based small RNA sequencing were performed at BMKGene (Münster, Germany), as previously described [40]. In short, small RNA sequencing was performed on an Illumina NovaSeq SP (Illumina, San Diego, CA, USA), with single-end 50 bp reads (SE50), generating final libraries comprising between 12 and 17 million clean reads per sample (Table 1).

**Table 1:** sRNA sequencing reads of replicate A and B

Replicate	Cell line	Virus	Clean reads	Total virus reads	% Viral clean reads	% Viral 21-nt	% Viral 24–29-nt
**A**	CPE/LULS50	WNV	14557753	985	0.01	42.34	6.40
	CPL/LULS56	WNV	12706582	2234	0.02	51.88	3.45
	CPE/LULS50	SINV	16284954	316,272	1.94	60.85	2.98
	CPL/LULS56	SINV	15,168432	177,180	1.17	60.70	2.96
	CPE/LULS50	BUNV (seg. L)	17,106,309	607,417	0.76	27.83	19.26
		BUNV (seg. M)		117,184	2.62	16.42	26.65
		BUNV (seg. S)		447,131	3.55	23.39	19.36
	CPL/LULS56	BUNV (seg. L)	14,558,210	518,494	0.80	25.87	20.18
		BUNV (seg. M)		985	3.07	16.32	25.65
		BUNV (seg. S)		2234	3.56	21.50	20.40
**B**	CPE/LULS50	WNV	28,867,020	794	2.75 × 10^–3^	18.77	4.41
	CPL/LULS56	WNV	16,288,959	949	0.01	25.08	5.27
	CPE/LULS50	SINV	16,498,489	3353	0.02	53.56	3.43
	CPL/LULS56	SINV	13,052,134	12,382	0.09	55.95	2.92
	CPE/LULS50	BUNV (seg. L)	15,916,137	124,485	0.78	70.52	62.60
		BUNV (seg. M)		310,881	1.95	14.65	24.32
		BUNV (seg. S)		432,849	2.72	5.89	6.65
	CPL/LULS56	BUNV (seg. L)	15,750,990	79,255	0.50	141.98	61.25
		BUNV (seg. M)		209,130	1.33	19.29	17.85
		BUNV (seg. S)		370,297	2.35	5.74	3.07

Resulting sequence reads were aligned to the respective virus reference genome (SINV GenBank accession number NC_001547, BUNV, all three viral segments, L, M and S; GenBank accession number NC_001927.1, NC_001926.1, NC_001925.1; WNV GenBank accession number MH924836.1) as previously described [13]. For virus discovery, small RNA reads were aligned to a curated database of all viral genomes, including reference flavivirus sequences from the NCBI Taxonomy Browser, the insect-associated viruses compiled from previously published research [19, 41–43] following established pipelines [16].

### Small RNA sequencing data availability

Small RNA sequencing data are available in the NCBI Sequence Read Archive under BioProject accession number PRJNA1346938.

## Results

### ISVs and arboviruses replicate in *Culex pipiens* cell lines

**Figure 1:**
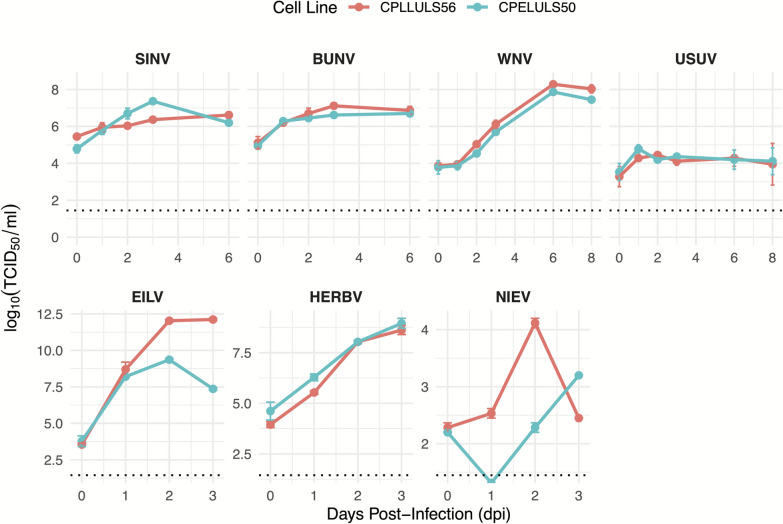
Virus replication kinetics in *Culex pipiens* cell lines CPE/LULS50 and CPL/LULS56. Cells were inoculated with seven different viruses: two alphaviruses (SINV, EILV), three flaviviruses (WNV, USUV, NIEV), or two peribunyaviruses (BUNV, HERBV), using an MOI 10 for the arboviruses (WNV, USUV, SINV, and BUNV) and MOI 1 for the ISVs (NIEV, EILV, and HERBV). The data points and error bars represent the means of three independent biological experiments and the standard error of the mean, respectively. The data were log-transformed and the mean and SEM of the log-transformed data are represented. The dotted line represents the limit of detection

To determine the susceptibility of the *Cx. pipiens* cell lines for different viruses, we infected CPE/LULS50 and CPL/LULS56 cells with representative arthropod-borne and insect-specific viruses belonging to the flaviviruses (WNV, USUV, NIEV), alphaviruses (SINV, EILV), and peribunyaviruses (BUNV, HERBV). All viruses successfully replicated in the cells, with the exception of NIEV, which produced low titer on both cell lines (Fig. 1). This demonstrates that CPE/LULS50 and CPL/LULS56 cells are broadly permissive to a wide spectrum of both arboviruses and ISVs across multiple virus families.

Both arthropod-borne (SINV) and insect-specific (EILV) alphavirus titers decreased in CPE/LULS50 post-peak, whereas in CPL/LULS56 cells titers maintained a plateau (Fig. 1). In the peribunyaviruses group, both arthropod-borne BUNV and insect-specific HERBV demonstrated an efficient replication profile in the two cell lines, with BUNV showing a slightly stronger replication in CPL/LULS56 (Fig. 1).

Within the flavivirus group, WNV and USUV exhibited robust replication in both cell lines with similar kinetics. However, NIEV, an insect-specific flavivirus, reached low titers and showed a marked delay in replication in CPE/LULS50 compared with CPL/LULS56 (Fig. 1).

### Both *Culex pipiens* cell lines have an active RNAi response

**Figure 2:**
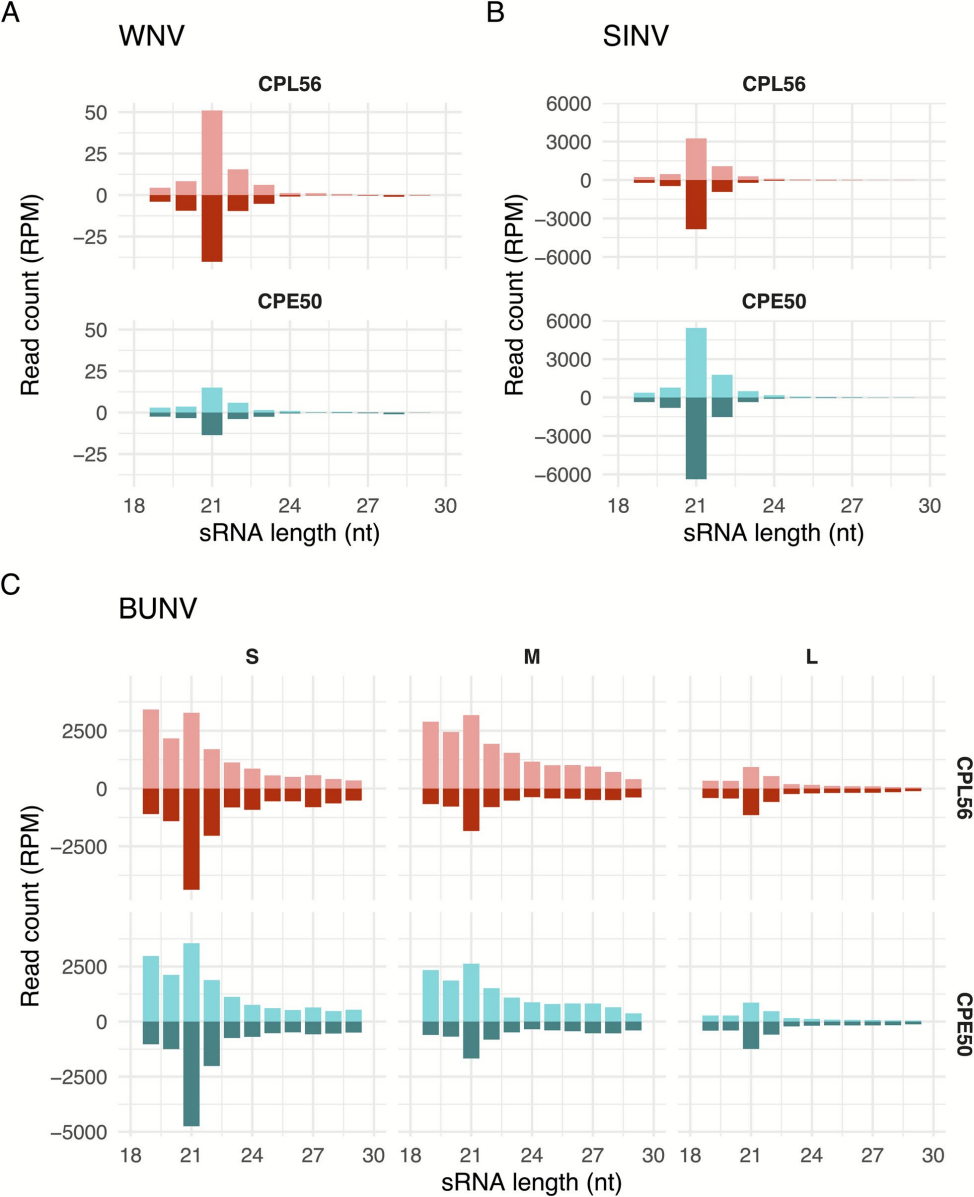
Replicate A small RNA length distribution in virus-infected CPE/LULS50 (CPE50) and CPL/LULS56 (CPL56) cells. **A** Positive strand WNV- and **B** positive strand SINV-specific small RNAs: positive numbers indicate mapping to the virus genome, and negative numbers indicate mapping to the antigenome. **C** Segmented negative strand BUNV- specific small RNAs: positive numbers indicate mapping to the BUNV antigenome, and negative numbers indicate mapping to the BUNV genome. RPM, reads per million total clean reads. Two independent biological experiments were carried out, replicate A is shown in the current figure. For replicate B, see Fig. S1

Small RNA sequencing was performed from CPE/LULS50 and CPL/LULS56 cells, in replicate A and B, infected with three different arboviruses, two positive strand RNA viruses (WNV and SINV), and one negative strand RNA virus (BUNV), to determine whether these cells exhibit an active RNAi response. In both cell lines, small RNAs derived from WNV, SINV, and BUNV were detected for replicate A (Table 1, Fig. 2) and replicate B (Table 1, Fig. S1). The lowest amount of virus-specific small RNAs were detected for WNV-infected cells. In both cell lines, small RNAs mapping to the viral genomes were predominantly 21nt long for SINV and WNV, but in case of BUNV, similar amounts of 21nt and longer (24-29nt long) small RNAs were detected, mapping to large, medium, and small (L, M and S) genome segments.

For all viruses, 21-nt vsiRNA reads mapped across the viral genomes in hot and cold spot patterns, with coverage of both genome and antigenome, and this pattern did not differ between cell types or between experimental replicates A (Fig. S2) and B (Fig. S3). For BUNV, the S segment was targeted more, compared with the M and L segments (Fig. 2C, Fig. S1C, Fig. S2A, Fig. S3A). For WNV, a prominent peak of antisense (v)siRNAs was detected in the 3′ untranslated region of the antigenome, coinciding with the known subgenomic flavivirus RNA (sfRNA) locus (Fig. S2B, Fig. S3B) [44]. For SINV, the 21-nt vsiRNA reads were distributed more evenly across the entire genome/antigenome compared with the other two viruses (Fig. S2C, Fig. S3C).

**Figure 3:**
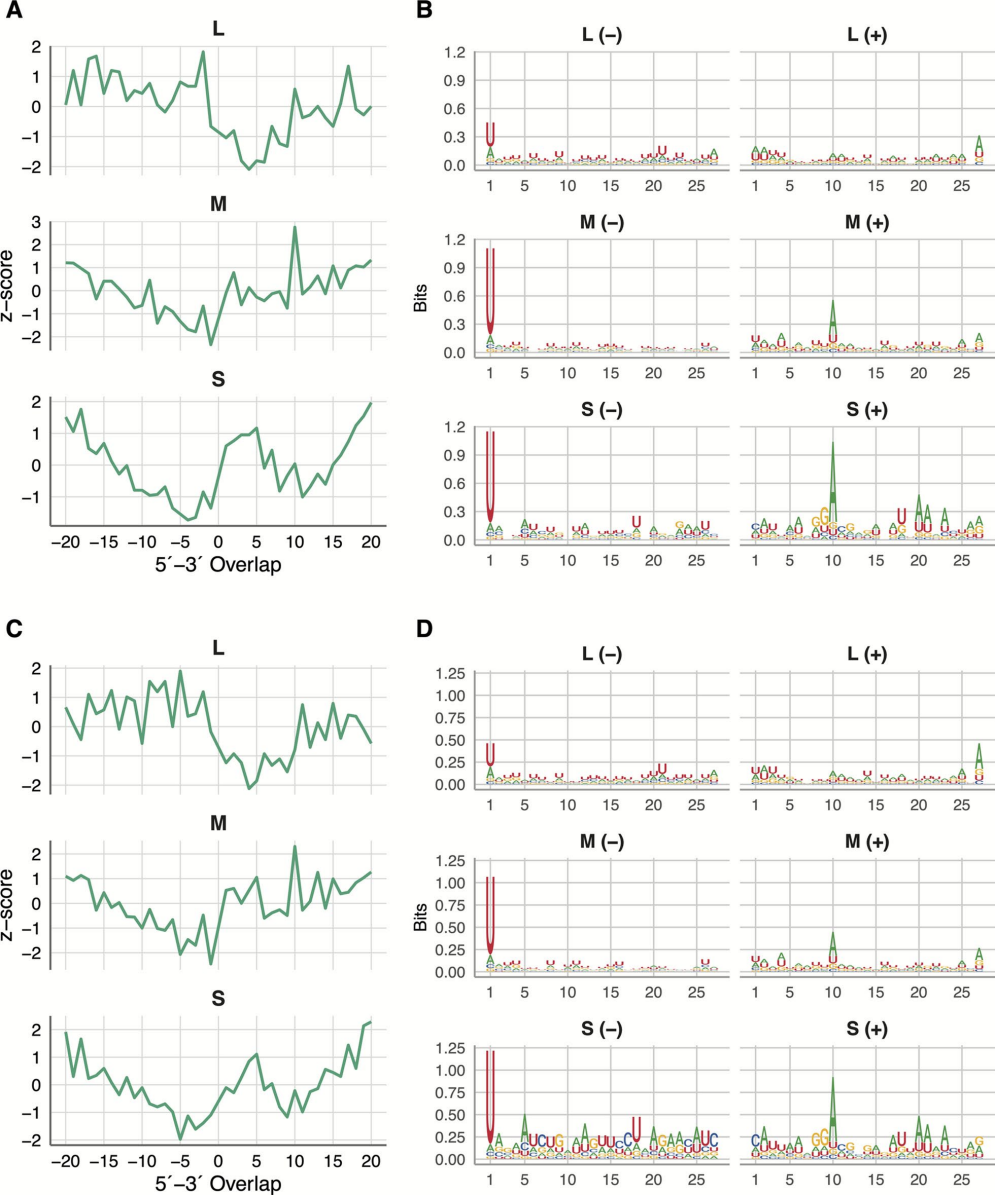
Replicate A ping-pong signature of piRNA-sized (24–29 nt) small RNAs produced during BUNV infection in CPE/LULS50 and CPL/LULS56 cells. Overlap *z*-score indicating the probability of overlap between the genome and antigenome of BUNV L, M, or S segment-specific piRNA-sized small RNAs in infected (**A**) CPE/LULS50 and (**C**) CPL/LULS56 cells; relative nucleotide frequency and conservation per position of BUNV L, M, or S segment-specific piRNA-sized small RNAs in infected (**B**) CPE/LULS50 and (**D**) CPL/LULS56 cells. Two independent biological experiments were carried out, and the results of replicate A are shown here. For replicate B, see Fig. S4

While piRNA-sized small RNAs (24–29 nt) were produced in both cell lines during infection with all tested viruses (Table 1), the characteristic ping-pong amplification signature of piRNA amplification was only present in BUNV-infected cells. The reads mapping to BUNV M and S genome segments displayed hallmarks of canonical piRNAs, including the ping-pong signature (10-nt overlap), and nucleotide biases (U1 and A10) in both cell lines for both replicate A (Fig. 3) and replicate B (Fig. S4). vpiRNAs induced by BUNV M and S segments generally mapped across the genome and antigenome for replicate A (Fig. S5A) and replicate B (Fig. S6A).

**Figure 4:**
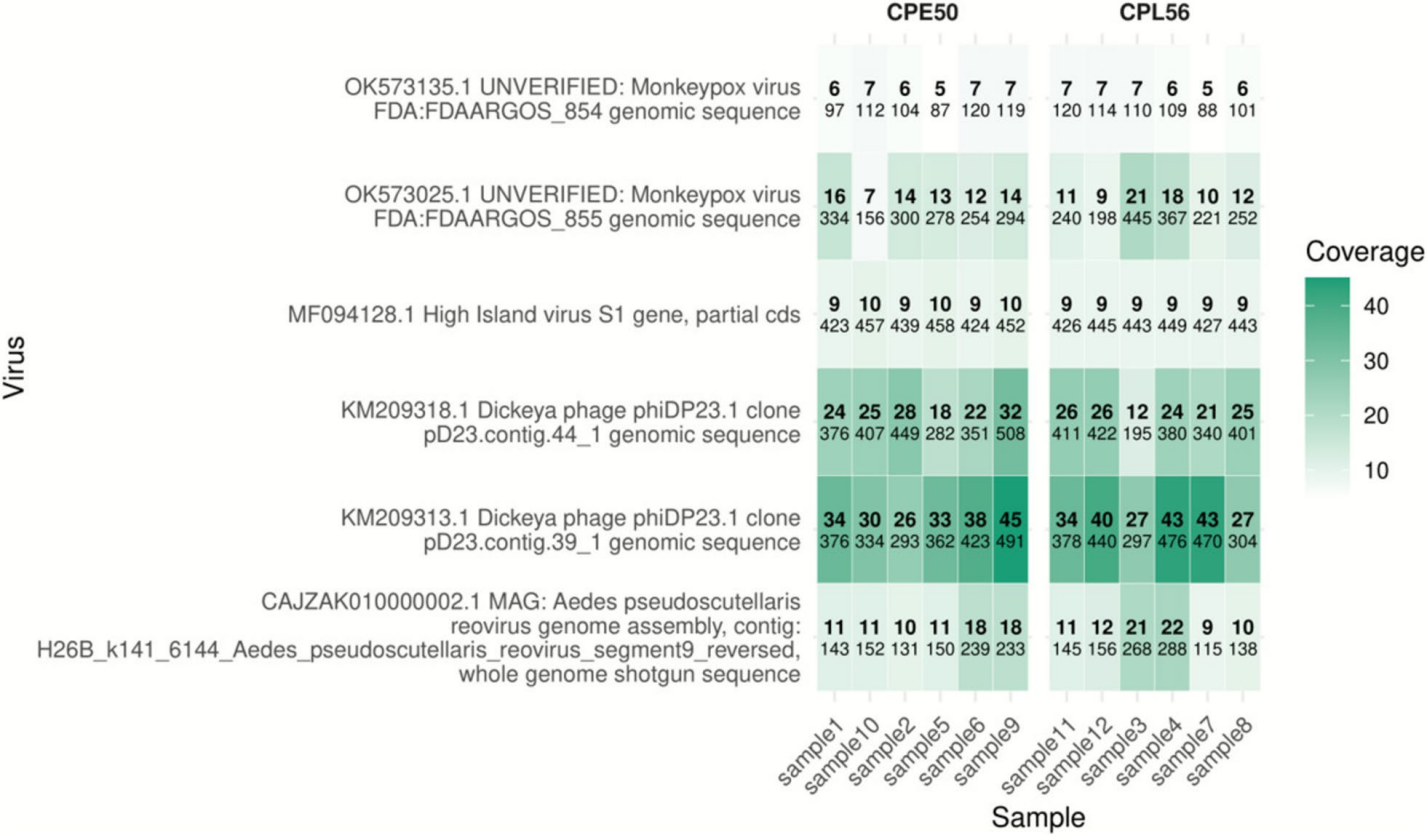
Heat map analysis shows potential persistent insect-specific virus diversity across CPE/LULS50 and CPL/LULS56 cell lines. Virus genome coverage by siRNAs (21nt) counts across CPE/LULS50 and CPL/LULS56 cell line samples for both replicate A and B. Only viral sequences detected in ≥ 3 samples are shown. Genome coverage (up to 45%) is represented by shading intensity, and siRNA read counts are indicated numerically within each cell

As shown previously, active virus replication induces the production of vsiRNAs, which normally map to at least 70% of the viral genome [16]. Therefore, to investigate potential ongoing insect-specific viral infections in the established cell lines, virus discovery analysis was performed as described previously [16] using the small RNA sequencing datasets. The 21nt siRNAs were mapped to a database with known viruses and the percentage coverage of these siRNAs to the tested viral genomes was determined. Several viral sequences were identified to which siRNAs mapped with a coverage of 9–40%, however, none were detected with a coverage of 70% or more (Fig. 4), suggesting an absence of active ISV replication in both cell lines. The lack of active ISV replication was further supported by immunofluorescence assays, in which no dsRNA staining was detected in the tested CPE/LULS50 cells, in contrast to the positive controls (Aag2 with persistent CFAV infection and Aag2 acutely infected with EILV) (Fig. S7).

## Discussion

*Culex pipiens* mosquitoes are widespread in Europe and increasingly implicated in the transmission of medically important viruses such as WNV, which progressively expanded into more northern regions marked by the dominance of lineage 2 over lineage 1a as the predominant circulating lineage, and USUV reported to now co-circulation with WNV across overlapping ecological niches from vector to avian hosts presenting a growing public health challenge [45, 46]. Understanding the vectors interactions with arboviruses and ISVs is critical for both surveillance and vector control; in this regard, cell culture systems play a pivotal role in advancing our understanding of virus–mosquito interactions. To evaluate two novel *Cx. pipiens ss* cell lines derived from European mosquitoes, CPE/LULS50 and CPL/LULS56, we characterized their permissiveness to viral infection and assessed their RNAi activity through small RNA sequencing.

Both cell lines supported replication of a broad spectrum of positive and negative strand arboviruses and ISVs, including SINV, BUNV, WNV, USUV, EILV, NIEV, and HERBV. This wide susceptibility is notable, especially given that other *Culex*-derived cell lines such as HSU have shown more restricted viral permissiveness [23]. While analyzed viruses exhibited overall similar replication kinetics in both lines, subtle differences were nonetheless observed. For instance, SINV replication appeared to drop faster in CPL/LULS56 compared with CPE/LULS50. Interestingly, WNV replicated robustly in both lines, while USUV and NIEV flaviviruses showed lower titers. These observations suggest that WNV may have replication strategies, allowing its robust replication in the cell lines that USUV and NIEV lack or that are not as effective.

Compared with commonly used *Aedes* spp. cell lines such as C6/36, which lacks a functional siRNA pathway [47], both *Cx. pipiens ss* lines exhibited robust siRNA responses, producing abundant viral-specific 21nt siRNAs across all tested viruses. This suggests the presence of a functional RNAi pathway and supports their utility in RNAi research. The siRNA responses were generally similar between the two cell lines. Notably, the siRNA profile during WNV infection revealed strong hot spots in the 3′ UTR of the antigenome, overlapping with the region producing sfRNA, suggesting targeted processing of this region [48]. Similar observations have been made in *Cx. pipiens* mosquitoes [21]. Furthermore, the amount of virus-specific small RNAs were lower for WNV-infected cells compared with SINV- or BUNV-infected cells. This could be due to the earlier timepoint chosen for BUNV/SINV versus WNV and the fact that WNV had not yet reached its peak at the chosen timepoint (96 hpi) in contrast to the other viruses, or that this is the result of WNV replication strategies to boycott the siRNA response, which could explain the relative success of WNV replication in these cell lines compared with the other two flaviviruses tested. However, differences in the amount of virus-specific small RNAs produced during infection between virus families have also been reported for other cell lines [21].

Analysis of piRNA-sized small RNAs revealed canonical vpiRNA production in BUNV-infected cells. These piRNAs originated from both the M and S segments and displayed typical ping-pong amplification signatures, which aligns with earlier findings in *Aedes* and some *Culex* cell lines [24, 40, 49]. In contrast, for WNV and SINV, piRNA-sized reads were only produced at low abundance and without canonical ping-pong amplification features, indicating limited piRNA pathway involvement, consistent with observations in *Cx. pipiens* mosquitoes and other *Culex* spp. cell lines [12, 22].

Investigating the presence of ISVs in mosquito cell lines is crucial, as latent or persistent infections can alter cellular physiology, affect experimental outcomes, and complicate the interpretation of virological studies. This consideration is particularly important when establishing new cell culture systems, since undetected ISV contamination may bias downstream applications and studies of virus–host interactions [50]. Previous investigations have shown that most mosquito cell lines harbor at least one, and often several, ISV infections [16, 21, 22, 51, 52]. Surprisingly, however, mapping the siRNAs to other known viruses for virus discovery did not suggest an active virus infection in these two *Culex pipiens*- derived cell lines. A reason for this could have been the presence of *Wolbachia* bacteria in these cell lines during their establishment, only later removed by antibiotic treatment [25]. *Wolbachia* are known to interfere with various RNA virus infections (including ISVs) [53–56].

Furthermore, we acknowledge the limitations in our detection method, as virus detection relied only on mapping viral siRNAs to deposited viral sequences. Therefore, unknown or weakly targeted viruses may have been missed. Moreover, the abundance of persistent viruses may vary over time, and our sampling may have coincided with a low replication phase for potential additional ISVs. Although this approach has been regularly successful in the past [16, 57–59], additional longitudinal and deeper metagenomic sequencing would be beneficial to confirm the results.

## Conclusions

Overall, these findings highlight the potential of CPE/LULS50 and CPL/LULS56 as virus-permissive and RNAi-competent mosquito cell lines. Despite the presence of a limited number of viral signatures, their consistent production of both (v)siRNAs and (v)piRNAs supports their utility for studying small RNA responses to viral infection. These features make them valuable tools for investigating *Culex*-borne virus–host interactions and the antiviral role of small RNAs, contributing to future efforts in understanding vector competence and developing potential RNAi-based vector control strategies.

## Supplementary Information

Additional file 1: Figure S1. Replicate B small RNA length distribution in virus-infected CPE/LULS50 (CPE50) and CPL/LULS56 (CPL56) cells. Figure S2. Replicate A distribution of viral specific 21 nt siRNAs across the viral genome and antigenome in infected *Culex pipiens* CPE/LULS50 and CPL/LULS56 cells. Figure S3. Replicate B distribution of viral specific 21 nt siRNAs across the viral genome and antigenome in infected *Culex pipiens* CPE/LULS50 and CPL/LULS56 cells. Figure S4. Replicate B ping-pong signature of piRNA-sized (24–29 nt) small RNAs produced during BUNV infection in CPE/LULS50 and CPL/LULS56 cells. Figure S5. Replicate A distribution of viral specific 28 nt piRNA-sized RNAs across the viral genome and antigenome in infected *Culex pipiens* CPE/LULS50 and CPL/LULS56 cells. Figure S6. Replicate B distribution of viral specific 28 nt piRNA-sized RNAs across the viral genome and antigenome in infected *Culex pipiens* CPE/LULS50 and CPL/LULS56 cells. Figure S7. 3G1 immunofluorescence assay for dsRNA detection in different cell lines.

## Data Availability

Small RNA sequencing data are available in the NCBI Sequence Read Archive under BioProject accession number PRJNA1346938.

## Abbreviations

FBS: Fetal bovine serum
RNAi: RNA interference
siRNA: Small interfering RNA
piRNA: PIWI-interacting small RNA
sRNA: Small RNA
vsiRNA: Viral siRNA
ISV: Insect-specific virus
