# Supplementary material for: Characterization of Culex pipiens cell lines: virus infection and RNAi response
Source: Parasit Vectors. 2026 Jan 28;19:94. doi: 10.1186/s13071-026-07248-w (PMC12924493; doi:10.1186/s13071-026-07248-w)
Supplement: Supplementary file 1 — Additional file 1: Figure S1. Replicate B small RNA length distribution in virus-infected CPE/LULS50 (CPE50) and CPL/LULS56 (CPL56) cells. Figure S2. Replicate A distribution of viral specific 21 nt siRNAs across the viral genome and antigenome in infected Culex pipiens CPE/LULS50 and CPL/LULS56 cells. Figure S3. Replicate B distribution of viral specific 21 nt siRNAs across the viral genome and antigenome in infected Culex pipiens CPE/LULS50 and CPL/LULS56 cells. Figure S4. Replicate B ping-pong signature of piRNA-sized (24–29 nt) small RNAs produced during BUNV infection in CPE/LULS50 and CPL/LULS56 cells. Figure S5. Replicate A distribution of viral specific 28 nt piRNA-sized RNAs across the viral genome and antigenome in infected Culex pipiens CPE/LULS50 and CPL/LULS56 cells. Figure S6. Replicate B distribution of viral specific 28 nt piRNA-sized RNAs across the viral genome and antigenome in infected Culex pipiens CPE/LULS50 and CPL/LULS56 cells. Figure S7. 3G1 immunofluorescence assay for dsRNA detection in different cell lines. [file 13071_2026_7248_MOESM1_ESM.docx]

**Supplementary Material**

**Characterization of *Culex pipiens* cell lines: virus infection and RNAi response**

Sarah Gothe et al.

Supplementary Fig1


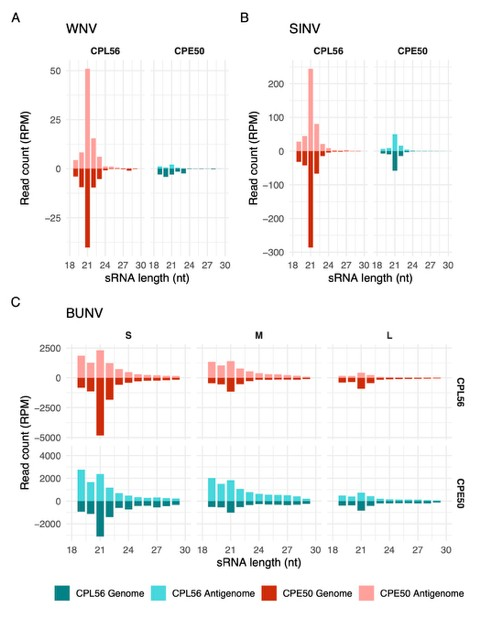


**Figure S1. Replicate B small RNA length distribution in virus-infected CPE/LULS50 (CPE50) and CPL/LULS56 (CPL56) cells.** (A) Positive strand WNV- and (B) positive strand SINV–specific small RNAs: positive numbers indicate mapping to the virus genome, and negative numbers indicate mapping to the antigenome. (C) Segmented negative strand BUNV– specific small RNAs: positive numbers indicate mapping to the BUNV antigenome, and negative numbers indicate mapping to the BUNV genome. RPM: Reads per million total clean reads. Two independent biological experiments were carried out, and the results of replicate B are shown here.

Supplementary Fig2

**L**

**M**

**S**


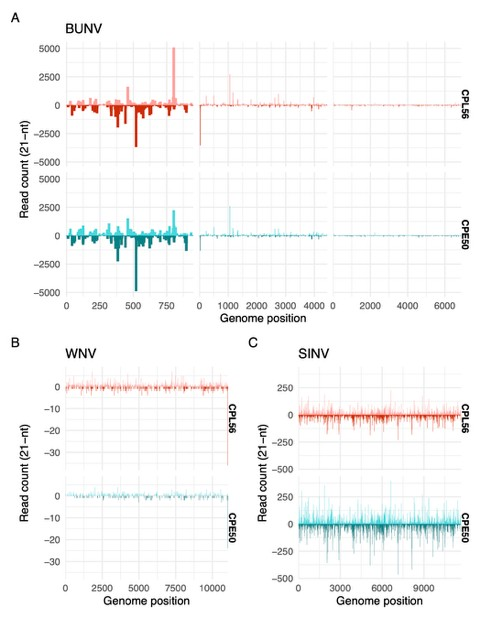


**Figure S2. Replicate A distribution of viral specific 21 nt siRNAs across the viral genome and antigenome in infected *Culex pipiens* CPE/LULS50 and CPL/LULS56 cells.** BUNV (A), WNV (B) and SINV (C)–specific siRNAs mapped along the genome and antigenome. For the positive strand WNV and SINV, positive numbers indicate mapping to the virus genome, and negative numbers indicate mapping to the antigenome. For the negative strand BUNV – specific siRNA mapping to the three segments (L, M and S), positive numbers indicate mapping to the virus antigenome and negative numbers indicate mapping to the BUNV genome. Two independent biological experiments were carried out for each experimental condition; plots from replicate A is presented here.

Supplementary Fig3


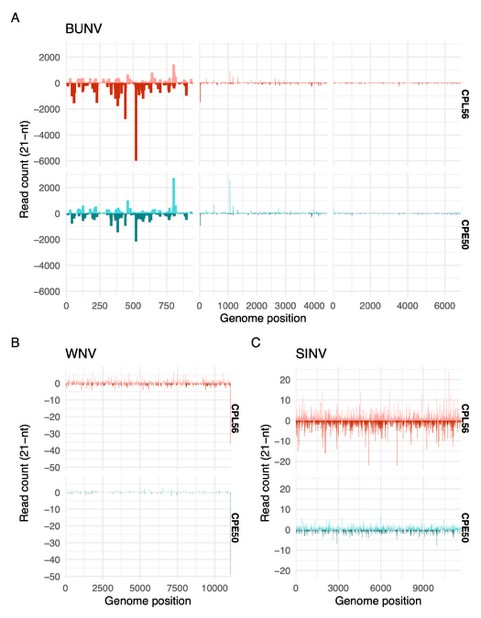


**M**

**L**

**S**

**Figure S3. Replicate B distribution of viral specific 21 nt siRNAs across the viral genome and antigenome in infected *Culex pipiens* CPE/LULS50 and CPL/LULS56 cells.** BUNV (A), WNV (B) and SINV (C)–specific siRNAs mapped along the genome and antigenome. For the positive strand WNV and SINV, positive numbers indicate mapping to the virus genome, and negative numbers indicate mapping to the antigenome. For the negative strand BUNV – specific siRNA mapping to the three segments (L, M and S), positive numbers indicate mapping to the virus antigenome and negative numbers indicate mapping to the BUNV genome. Two independent biological experiments were carried out for each experimental condition; plots from replicate B are presented here.

Supplementary Fig4


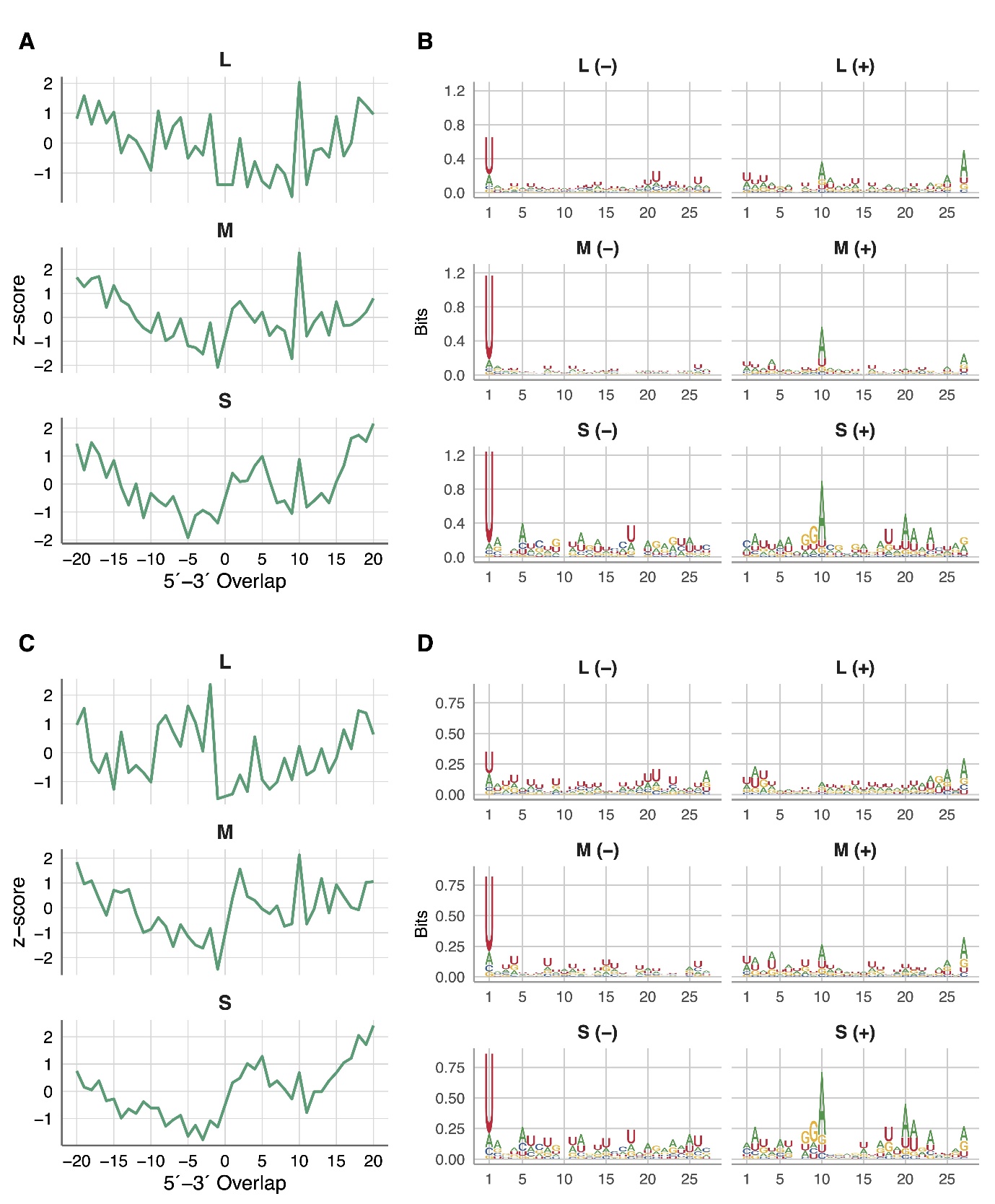


**Figure S4. Replicate B ping-pong signature of piRNA-sized (24–29 nt) small RNAs produced during BUNV infection in CPE/LULS50 and CPL/LULS56 cells.** Overlap z-score indicating the probability of overlap between the genome and antigenome of BUNV L, M or S segment-specific piRNA-sized small RNAs in infected (A) CPE/LULS50 and (C) CPL/LULS56 cells; relative nucleotide frequency and conservation per position of BUNV L, M or S segment-specific piRNA-sized small RNAs in infected (B) CPE/LULS50 and (D) CPL/LULS56 cells. Two independent biological experiments were carried out, and the results of replicate B are shown here.

Supplementary Fig5


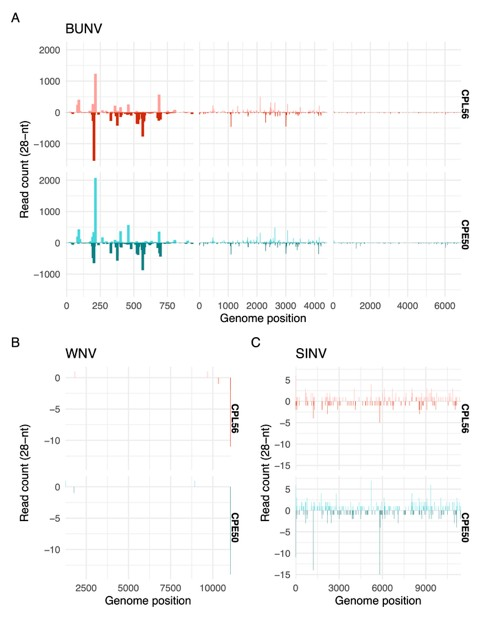


**L**

**M**

**S**

**Figure S5. Replicate A distribution of viral specific 28 nt piRNA-sized RNAs across the viral genome and antigenome in infected *Culex pipiens* CPE/LULS50 and CPL/LULS56 cells.** BUNV (A), WNV (B) and SINV (C)–specific 28 nt RNAs mapped along the genome and antigenome. For the positive strand WNV and SINV, positive numbers indicate mapping to the virus genome, and negative numbers indicate mapping to the antigenome. For the negative strand BUNV – specific siRNA mapped to all three segments (L, M and S), positive numbers indicate mapping to the virus antigenome and negative numbers indicate mapping to the BUNV genome. Two independent biological experiments were carried out for each experimental condition; plots from replicate A are presented here.

Supplementary Fig6


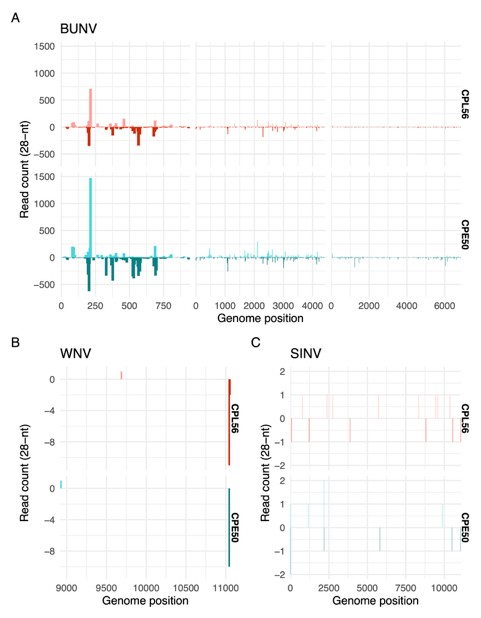


**S**

**M**

**L**

**Figure S6. Replicate B distribution of viral specific 28 nt piRNA-sized RNAs across the viral genome and antigenome in infected *Culex pipiens* CPE/LULS50 and CPL/LULS56 cells.** BUNV (A), WNV (B) and SINV (C)–specific 28 nt RNAs mapped along the genome and antigenome. For the positive strand WNV and SINV, positive numbers indicate mapping to the virus genome, and negative numbers indicate mapping to the antigenome. For the negative strand BUNV – specific siRNA mapped to all three segments (L, M and S), positive numbers indicate mapping to the virus antigenome and negative numbers indicate mapping to the BUNV genome. Two independent biological experiments were carried out for each experimental condition; plots from replicate B are presented here.

Supplementary Fig7

**A**


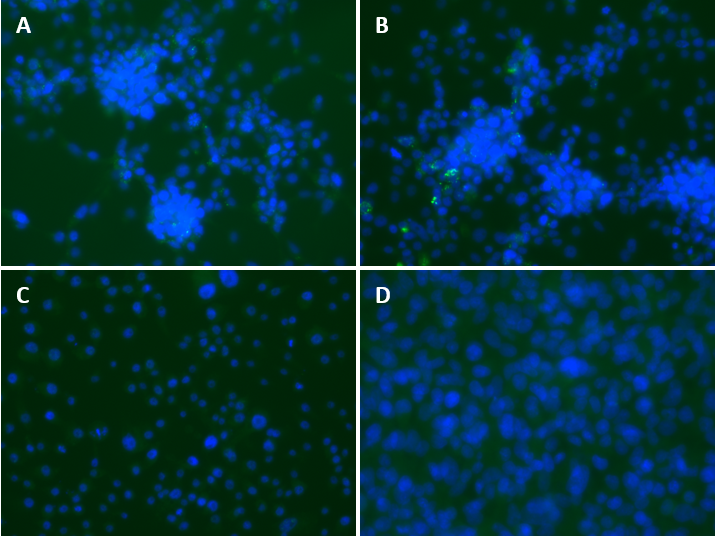


**Figure S7. 3G1 immunofluorescence assay for dsRNA detection in different cell lines.**

Immunofluorescence assay performed on *Ae. aegypti*-derived Aag2 persistently infected with Cell fusing agent virus (A), Aag2 infected with Eilat virus MOI 1 and fixed at 24 hours post infection as positive control for the detection of viral dsRNA by mAb 3G1 (B), CPE/LULS50 (C), BHK-21 (D). Each panel shows a representative image for its respective experimental condition. Cells were fixed by paraformaldehyde and incubated with dsRNA-specific mAb 3G1, followed by goat anti-mouse Alexafluor 488 (green). DNA are labelled with DAPI stain (blue). Plates were imaged at 40x magnification. Scale bar denotes 100 μm.
